# A Novel Linear B‐Spline Mixed Effect Model for Alzheimer’s Disease Clinical Study Data

**DOI:** 10.1002/alz70859_099565

**Published:** 2025-12-25

**Authors:** Kun Jin, Guoqiao Wang

**Affiliations:** ^1^ Anavex Life Sciences Corp., New York, NY USA; ^2^ Washington University in St. Louis, School of Medicine, St. Louis, MO USA

## Abstract

**Background:**

Mixed models for repeated measures (MMRM) are widely used as primary analysis models in Alzheimer’s disease and other neurological clinical trials due to their robust statistical properties. A central aspect of these models is treating study visits as categorical variables, which limits their application in clinical trial data. Specifically, MMRM cannot accommodate unscheduled visits, leading to a loss of information and increased missing data. Additionally, integrative studies cannot utilize these models for trials with different visit schedules. Lastly, as more wireless and densely spaced data become available, MMRM is not suitable for such data. This study proposes a novel linear B‐spline MMRM (LB‐MMRM) using time as a continuous variable and evaluates its performance against traditional MMRM and cubic spline MMRM methods.

**Method:**

The LB‐MMRM incorporates linear B‐Spline bases, treating visits as continuous variables. This approach allows the model to include unscheduled visits, making it suitable for integrative study analysis, even when trials have different visit schedules. As a parametric model for longitudinal data, it provides a foundation for handling continuous wireless data. The model will be compared to standard MMRM and the cubic spline model using simulated data that mimic disease progression patterns of CDR‐SB and ADAS‐Cog observed in the Clarity‐AD and TRAILBLAZER‐ALZ 2 trials. The proportional treatment effects will be incorporated into these models to evaluate treatment efficacy over the follow‐up period.

**Result:**

When only scheduled visit data are used, the proposed LB‐MMRM and common MMRM generate the same results. Additionally, it simplifies the estimation of overall treatment effects and offers a more intuitive interpretation than cubic spline models. Comprehensive simulation results will demonstrate the relative performance of these models.

**Conclusion:**

The LB‐MMRM is a natural extension of traditional MMRM for regulatory review and clinical application. Its ability to incorporate unscheduled visits, integrate studies with varying visit schedules, and provide intuitive and straightforward implementation makes it a promising alternative to existing models. This approach addresses key limitations in longitudinal clinical trial analyses.

